# Supplementary figures and images for: Advanced radiotherapy technique in hepatocellular carcinoma with portal vein thrombosis: Feasibility and clinical outcomes
Source: PLoS One. 2021 Sep 23;16(9):e0257556. doi: 10.1371/journal.pone.0257556 (PMC8460041; doi:10.1371/journal.pone.0257556)

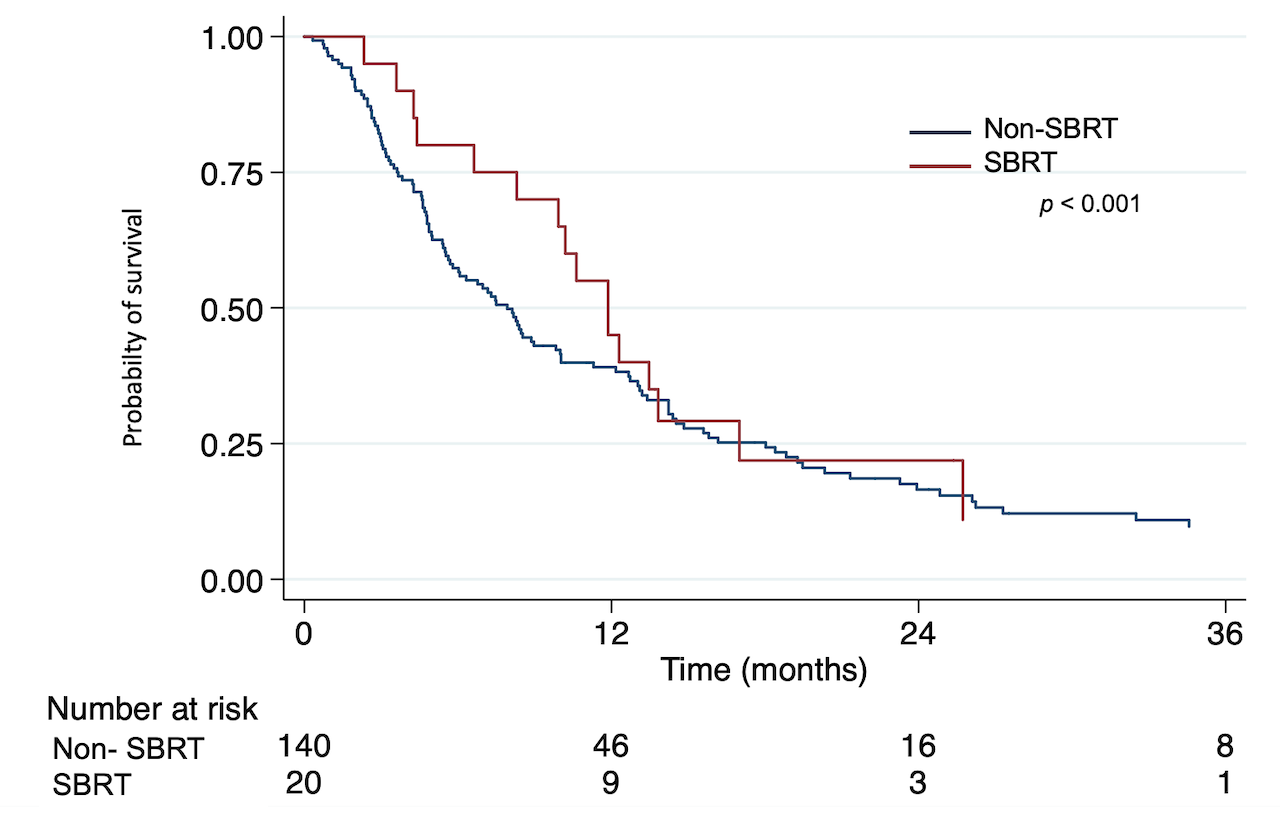

Supplement: S1 Fig — (TIF) [file pone.0257556.s001.tif]
