# Supplementary material for: Advanced radiotherapy technique in hepatocellular carcinoma with portal vein thrombosis: Feasibility and clinical outcomes
Source: PLoS One. 2021 Sep 23;16(9):e0257556. doi: 10.1371/journal.pone.0257556 (PMC8460041; doi:10.1371/journal.pone.0257556)
Supplement: S1 Table — (DOCX) [file pone.0257556.s002.docx]

**S1 Table. Comparison of patient and treatment characteristics between 2007 to 2014 and 2015 to 2019**

|  | **2007-2014** | **2015-2019** |
| --- | --- | --- |
| Number | 40 (25%) | 120 (75%) |
| Age (mean) | 55.9 | 61.5 |
| Child-Pugh score  5-6  7-9  10-15  Missing | 30 (75%)  9 (22.5%)  1 (2.5%)  0 | 79 (65.8%)  36 (30%)  3 (2.5%)  2 (1.7%) |
| Tumor size (cm)  Median (range)  <10 cm  ≥10cm  Missing | 5.6 (1-22.5)  31 (77.5%)  8 (20%)  1 (2.5%) | 8.7 (1.2-24.5)  63 (52.5%)  57 (47.5%)  0 |
| Site of PVTT  Main or bilateral portal vein  Unilateral portal vein  Others  Missing | 24 (60%)  11 (27.5%)  5 (12.5%)  0 | 61 (50.8%)  40 (33.3%)  15 (12.5%)  4 (3.3%) |
| Radiation technique  3D-CRT  IMRT/VMAT  SBRT | 31 (77.5%)  7 (17.5%)  2 (5%) | 9 (7.5%)  93 (77.5%)  18 (15%) |
| BED (Gy_10_)  Mean  < 56 Gy_10_  ≥ 56 Gy_10_ | 51.6  22 (55%)  18 (45%) | 49.0  91 (75.8%)  29 (24.2%) |
