# Supplementary material for: Advanced radiotherapy technique in hepatocellular carcinoma with portal vein thrombosis: Feasibility and clinical outcomes
Source: PLoS One. 2021 Sep 23;16(9):e0257556. doi: 10.1371/journal.pone.0257556 (PMC8460041; doi:10.1371/journal.pone.0257556)
Supplement: S2 Table — (DOCX) [file pone.0257556.s003.docx]

**S2 Table.** **Univariate analysis of potential predictive factors for any grade 3/4 liver toxicities**

| **Variables** | **Univariate analysis** | | |
| --- | --- | --- | --- |
|  | **OR** | **95% CI** | **P-value** |
| Age | 1.01 | 0.98 – 1.04 | 0.46 |
| Child-Pugh score (B or C vs A) | 1.85 | 0.83 – 4.12 | 0.13 |
| Hepatitis B infection (yes vs no) | 0.97 | 0.42 – 2.10 | 0.94 |
| Hepatitis C infection (yes vs no) | 1.51 | 0.65 – 3.55 | 0.34 |
| Cirrhosis (yes vs no) | 1.06 | 0.25 – 5.54 | 0.94 |
| Main PVTT location (yes vs no) | 1.53 | 0.70 – 3.35 | 0.29 |
| Tumor size (cm) | 0.95 | 0.88 – 1.04 | 0.27 |
