# Supplementary material for: Advanced radiotherapy technique in hepatocellular carcinoma with portal vein thrombosis: Feasibility and clinical outcomes
Source: PLoS One. 2021 Sep 23;16(9):e0257556. doi: 10.1371/journal.pone.0257556 (PMC8460041; doi:10.1371/journal.pone.0257556)
Supplement: S3 Table — (DOCX) [file pone.0257556.s004.docx]

**S3 Table. Comparison of patient and treatment characteristics between SBRT and Non-SBRT groups.**

|  | **SBRT** | **Non-SBRT** |
| --- | --- | --- |
| Number | 20 (12.5%) | 140 (87.5%) |
| Age (mean) | 55.9 | 61.5 |
| Child-Pugh score  5-6  7-9  10-15  Missing | 16 (80%)  4 (20%)  0  0 | 93 (66.4%)  41 (29.2%)  3 (7.5%)  2 (7.5%) |
| Tumor size (cm)  Median (range)  <10 cm  ≥10cm  Missing | 3.9 (1.7-18.4)  18 (90%)  2 (10%)  0 | 8.5 (1-24.5)  76 (54.3%)  63 (45%)  1 (0.7%) |
| Site of PVTT  Main or bilateral portal vein  Unilateral portal vein  Others  Missing | 9 (45%)  7 (35%)  4 (20%)  0 | 76 (54.3%)  44 (31.4%)  16 (11.4%)  4 (2.9%) |
| BED (Gy_10_)  Mean  < 56 Gy_10_  ≥ 56 Gy_10_ | 75.9  5 (25%)  15 (75%) | 45.8  108 (77.1%)  32 (22.9%) |
