# Supplementary material for: Advanced radiotherapy technique in hepatocellular carcinoma with portal vein thrombosis: Feasibility and clinical outcomes
Source: PLoS One. 2021 Sep 23;16(9):e0257556. doi: 10.1371/journal.pone.0257556 (PMC8460041; doi:10.1371/journal.pone.0257556)
Supplement: S4 Table — (DOCX) [file pone.0257556.s005.docx]

**S4 Table Survival data comparison between SBRT and Non-SBRT groups**

|  | **SBRT** | **Non-SBRT** | **P-Value** |
| --- | --- | --- | --- |
| **Median Survival (months)** | 11.9 | 7.9 | 0.51 |
| **1-year overall survival (%)** | 45 | 39.1 |  |
| **2-year overall survival (%)** | 22 | 16.5 |  |
