# Supplementary material for: Advanced radiotherapy technique in hepatocellular carcinoma with portal vein thrombosis: Feasibility and clinical outcomes
Source: PLoS One. 2021 Sep 23;16(9):e0257556. doi: 10.1371/journal.pone.0257556 (PMC8460041; doi:10.1371/journal.pone.0257556)
Supplement: S5 Table — (DOCX) [file pone.0257556.s006.docx]

**S5 Table The median survival time and objective response rate in studies employing stereotactic body radiotherapy in patients with HCC with PVTT**

| Studies | Year | Design | No | Treatment | Radiation Dose (Median) | OR | Median OS (months) |
| --- | --- | --- | --- | --- | --- | --- | --- |
| Xi (11) | 2013 | Retrospective | 41 | SBRT | 36Gy/6Fx | 75.6% | 13 |
| Kang (12) | 2014 | Retrospective | 101 | SBRT ± TACE | 40.2Gy/6Fx | 70.2% (PVTT)  87.1% (Tumor) | 12-15 |
| Matsuo (13) | 2016 | Retrospective | 43 | SBRT | BED 73.4 Gy_10_ | 67% | 11 |
| Shui (14) | 2018 | Retrospective | 70 | SBRT ± TACE | 40Gy/5Fx | 79.1% | 10 |
| Choi (15) | 2020 | Retrospective | 24 | SBRT ± TACE | 45Gy/3Fx | 54.1% | 20.8 |
| This study | 2021 | Retrospective | 20 | SBRT ± TACE | 45Gy/5Fx | 80% | 11.9 |
